# Selection of WHO-recommended essential medicines for non-communicable diseases on National Essential Medicines Lists

**DOI:** 10.1371/journal.pone.0220781

**Published:** 2019-08-09

**Authors:** Jordan D. Jarvis, Hannah Woods, Anjli Bali, Efosa Oronsaye, Nav Persaud

**Affiliations:** 1 London School of Hygiene & Tropical Medicine, London, England, United Kingdom; 2 Centre for Urban Health Solutions, St. Michael’s Hospital, Toronto, Ontario, Canada; 3 Department of Family and Community Medicine, St. Michael’s Hospital, Toronto, Ontario, Canada; Uppsala University, SWEDEN

## Abstract

**Background:**

Non-communicable diseases (NCDs) are the leading cause of death worldwide. Inadequate and inequitable access to essential NCD medicines is a major concern, particularly in low- and middle-income countries. National Essential Medicines Lists (EMLs) are important policy tools that indicate which medicines are prioritized as essential within a country’s health system. This study sought to analyze a wide range of national essential medicines lists (EMLs) for their inclusion of priority non communicable disease (NCD) interventions recommended by the World Health Organization (WHO).

**Methods:**

Three lists of WHO endorsed priority NCD interventions were included. A database with 137 national EMLs and the WHO EML was created from the WHO Repository and these EMLs were compared for listing of priority NCD interventions.

**Results:**

Across 137 countries with national EMLs, the median percentage of 20 Best Buys interventions listed was 90% (IQR 80–95) and 31 Package of essential noncommunicable disease interventions (PEN) interventions listed was 94% (IQR 90–97), of 9 HEARTS interventions was 100% (IQR 89–100), and of the 43 unique interventions across the three priority lists was 88% (IQR 84–93). Less than 80% of the 43 interventions were listed by 22 (16%) countries and less than half of the interventions were listed by 2 countries: Angola (35%) and Cambodia (23%). Interventions listed on the fewest number of national EMLs were: influenza vaccine, HPV vaccine, hepatitis B vaccine, cervical cancer chemotherapy, codeine, promethazine, senna, and oxygen.

**Conclusion:**

Most NCD interventions have been prioritized in national policy in most cases. The majority of priority medicines for NCDs described within key WHO NCD technical packages are listed on nearly all national EMLs across 137 countries of all income levels. Most NCD interventions have been prioritized in national policy in most cases, but in some countries and for select interventions such as the HPV vaccine, prioritization may be reviewed.

## Background

Non-communicable diseases (NCDs), which include cardiovascular disease, cancer, chronic respiratory disease, and diabetes are responsible for 71% of deaths worldwide [1]. The burden of morbidity and mortality from NCDs disproportionately affects individuals living in low- or middle-income countries (LMICs) where 67% of NCD deaths occur. In 2015, governments committed to working toward progress on achieving Sustainable Development Goal 3 to promote health and well-being, which prioritizes a 30% global reduction of NCDs and the achievement of universal health coverage including access to essential medicines for all. Governments also agreed to the World Health Organization’s (WHO) 9 global targets aimed at addressing NCDs; two of which are to: “have at least 50% of eligible people receive drug therapy and counselling (including glycemic control) to prevent heart attacks and strokes” and “achieve 80% availability of affordable basic technologies and essential medicines to treat major NCDs” [2]. A baseline study on the latter showed that across low, lower-, and upper-middle income countries only 15%, 23%, and 36% of lowest-priced generics met the target with overall comparable levels in public and private facilities [3]. Global disparities in access to basic medications for NCDs exceed those for acute conditions particularly across LMICs [4].

Accounting for a quarter of all health expenditures globally, access to affordable essential medicines represent an important policy issue for countries of all income levels [5, 6]. The WHO Model Essential Medicines List (EML) serves as an international evidence-based guide of clinically important interventions which countries often use to formulate their own national essential medicines lists (EMLs) that will meet the priority health needs of their populations [7]. The selection of medicines on national EMLs are thought to be an important initial step toward making medicines available as these lists often serve for the basis for medicine procurement and reimbursement within a country. Studies indicate that medicines on national EMLs are more frequently available and affordable than those not listed on national EMLs [8].

The WHO has prioritized certain interventions and guidance for their implementation toward the prevention and control of NCDs within the WHO Global NCD Action Plan and other technical documents [2]. To our knowledge, there has been no comprehensive study comparing WHO-recommended priority NCD medicines listed across national EMLs. This analysis sought to determine across a wide range of countries which national EMLs have listed the priority NCD interventions recommended by WHO NCD packages and guidelines.

## Method

The 137 countries included in the analysis were the only countries with a publicly available essential medicines list [9].

### Priority NCD intervention list selection

We selected three lists of priority NCD interventions for the prevention and management of NCDs that have been endorsed by the WHO: the Tackling NCDs “Best Buys” publication (Objective 4; also known as Appendix 3 of the WHO Global Action Plan for the Prevention and Control of NCDs 2013–2020) [10], the Package of Essential Noncommunicable Disease Interventions for Primary Health Care (PEN; 2010) [11], and the HEARTS Technical Package for Cardiovascular Disease Management in Primary Health Care (HEARTS; 2016) [12]. The purpose, scope, brief description of process to develop the documents, and the year published are summarized for each priority intervention list in Table 1.

**Table 1:** WHO NCD priority list characteristics.

	Purpose	Scope	Process	Date published/updated
Best Buys (Objective 4) (Also known as “Appendix 3” of the WHO Global Action Plan on NCDs 2013–2020)	Cost-effective and recommended interventions that Member States can adopt to their national context as needed to implement measures towards achieving the Sustainable Development Goals (SDG) Target 3.4	Key NCD risk factors (tobacco, harmful use of alcohol, unhealthy diet and physical inactivity) and for cardiovascular disease, diabetes, cancer and chronic respiratory disease	Developed through technical experts meetings (with WHO conflicts of interest declaration) and adopted by Member States (MS) as Appendix 3 in 2014, with updates to Appendix 3 adopted by MS in 2017 (30)	Last updated 2017
Package of Essential Non-communicable Disease Interventions for Primary Health Care (PEN)	Defines a minimum set of essential NCD interventions for resource-poor settings to integrate and scale up in primary health care, in order to support universal coverage reforms toward stronger health systems	Heart disease, stroke, cardiovascular risk, diabetes, cancer, asthma and chronic obstructive pulmonary disease in primary health care	The WHO (former) Department of Chronic Disease Prevention and Management coordinated a multidisciplinary group of international experts across the field of NCDs and from diverse WHO Member States to produce PEN and for its peer review (with WHO conflicts of interest declaration)	2010
HEARTS Technical package for cardiovascular disease management in primary health care	Technical package to support Ministries of Health in improving cardiovascular disease management in primary health care settings	Cardiovascular disease prevention and management in primary care. Aligns with and builds on PEN	The HEARTS package is an expansion of the content on cardiovascular disease and diabetes from the WHO PEN documents. This package was developed through expert (WHO conflicts of interest declaration) consultation, engagement and review of evidence. The package was supported and cleared by partner organizations subject to clearance processes at WHO	2016

For each of the three lists of priority interventions, we generated a list of priority medicines by including the medicines or therapeutic classes mentioned in the document. When priority lists mentioned a therapeutic class rather than a particular medicine, we used the WHO 20^th^ Model EML to determine the medicines used to treat the condition [7].

### Data collection

Five country characteristics were collected for the 137 countries with national EMLs [13]: geographic region, population size, gross domestic product (GDP) per capita, publication date, and number of medicines on each national EML [see S1 Table]. Data on geographic region and health expenditure per capita was collected from the Global Health Observatory of the WHO (2018) using the most recent information available at the time [14]. We extracted data on population size and GDP per capita from the Central Intelligence Agency’s World Factbook (2018) [15].

Characteristics of Best Buys and PEN were obtained directly from the online documents and those of HEARTS were confirmed with relevant WHO staff [10–12].

We created a database of national EMLs by abstracting medicines listed on the most recent national EMLs posted on the WHO’s National Essential Medicines Lists Repository [9]. Medicines were listed individually using International Nonproprietary Names without regard for salts, doses, or formulations. Saline solutions, diagnostic agents, antiseptics, and disinfectants were excluded from the database [16]. We assumed that the medicines in the same chemical subgroup as the exemplar were equivalent (e.g. perindopril was considered equivalent to ramipril). All national EMLs were evaluated regardless of language of origin or publication date and a tally of all medications was done for individual lists [16]. We determined whether each national EML covered each priority medicine or class across the selected interventions. If the EML included at least one medicine within the priority class we considered that class covered.

Missing priority NCD medicines or therapeutic classes were identified from each national EML and sorted using the anatomical therapeutic chemical (ATC) code classification [17]. Basic statistical calculations were done using Microsoft excel.

#### Ethics approval

No ethics approval was sought for this review of publicly available information.

## Results

The 137 national EMLs (of 195 total countries; 70%) published between 2001 and 2016 have between 44 and 990 medicines listed (median 310) [see S1 Table].

### NCD “Best Buys” priority interventions

**Fig 1:**
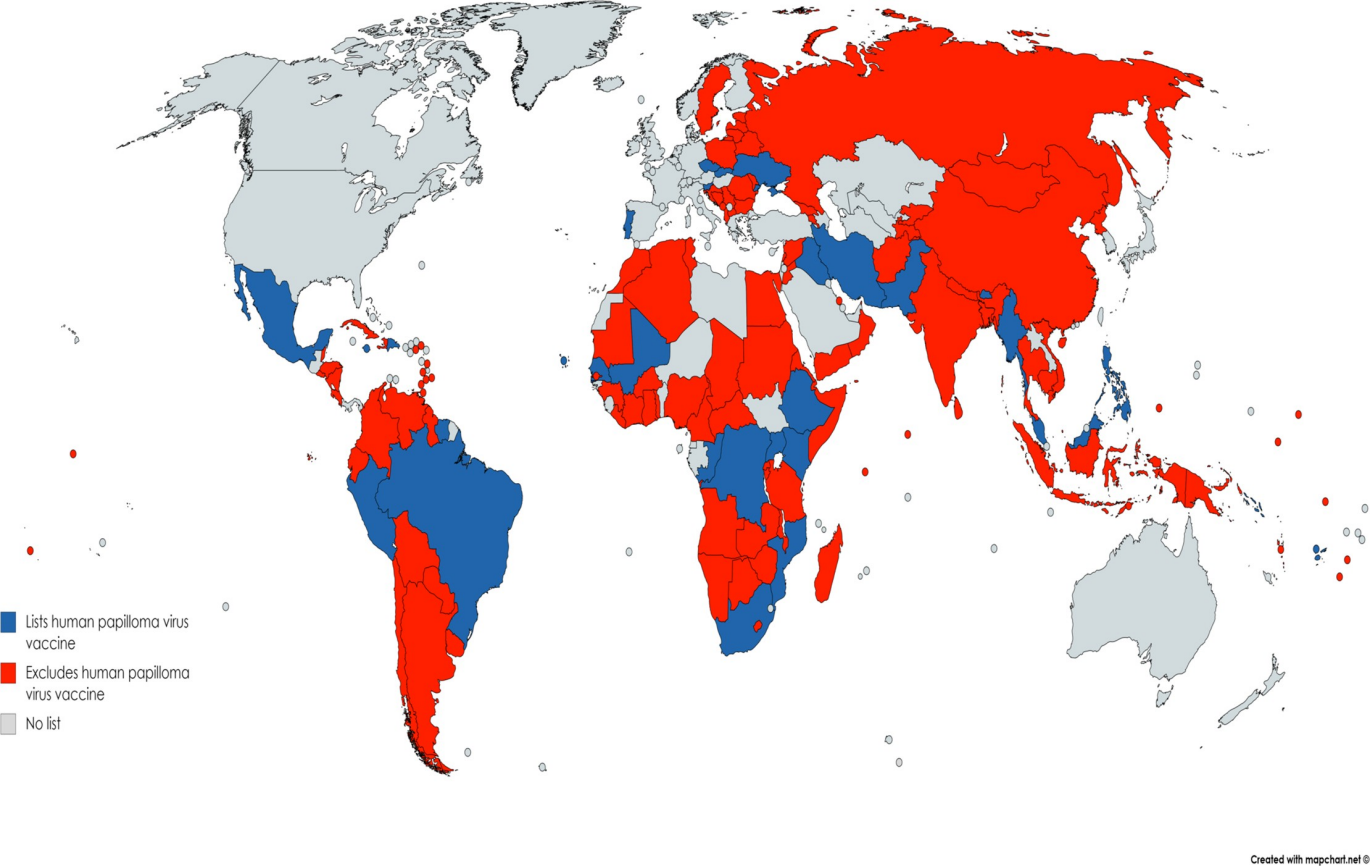
Countries that list or do not list the HPV vaccine on their national Essential Medicines List.

The median percentage of countries that included each of the 20 interventions recommended in the Best Buys document was 97% (133 of 137) (IQR: 81–99%); that is, Best Buys priority medicines were included in most national EMLs (Table 2). The priority medicines included in the fewest national EMLs were influenza vaccination (51 of 137 countries, 37%) and human papillomavirus (HPV) vaccination (30 of 137 countries, 22%) (Fig 1).

**Table 2:** Best Buys interventions and the number of countries that listed them on their national EML.

Condition	Intervention	# of countries listing (%)
***Cardiovascular disease***	Angiotensin converting enzyme inhibitor*	134 (98)
	Beta-blocker	136 (99)
	Diuretic	134 (98)
	Acetylsalicylic acid for ischemic stroke**	131 (96)
	Treatment of new cases of acute myocardial infarction with either: acetylsalicylic acid, or acetylsalicylic acid and clopidogrel, or thrombolysis	137 (100)
	Treatment of streptococcal pharyngitis	137 (100)
	Prophylactic penicillin for secondary prevention of rheumatic fever and rheumatic heart disease	137 (100)
	Treatment of acute ischemic stroke with intravenous thrombolytic therapy	87 (64)
	Anticoagulation for medium-and high-risk non-valvular atrial fibrillation and for mitral stenosis with atrial fibrillation	111 (81)
***Manage diabetes***	Drug therapy (including glycaemic control for diabetes mellitus) (Oral Hypoglycemics)	136 (99)
	Insulin	135 (99)
	Influenza Vaccination*	51 (37)
***Cancer***	HPV vaccination	30 (22)
	Prevention of liver cancer through hepatitis B immunization	109 (80)
	Colorectal cancer chemotherapy	116 (85)
	Cervical cancer chemotherapy (cisplatin)	91 (66)
	Breast cancer chemotherapy	131 (96)
	Opiates for basic palliative care	133 (97)
***Chronic respiratory disease***	Symptom relief for patients with asthma with inhaled salbutamol/ Symptom relief for patients with chronic obstructive pulmonary disease with inhaled salbutamol	135 (99)
	Treatment of asthma using low dose inhaled beclomethasone and short acting beta agonist	136 (99)
***Median % (IQR)***		**97 (81–99)**

*ACE inhibitors are listed for both cardiovascular disease and diabetes management, and influenza vaccination is listed for treatment of diabetes and for chronic respiratory disease; we have included them once to avoid repetition.

** Regardless of acetylsalicylic acid dosage

The median percentage of Best Buys priority interventions included by each country was 90% (18 of 20) (IQR: 80–95%); that is, EMLs included most priority medicines for primary care (Table 3). Thirteen countries listed all 20 Best Buys interventions on their EML (Table 3).

**Table 3:** The number and percentage of Best Buys, PEN, and HEARTS interventions listed on national EMLs.

Country	Best Buys (%) (n = 20)	PEN (%) (n = 31)	HEARTS (%) (n = 9)	Total unique priority interventions (%) (n = 43)
**Afghanistan**	15 (75)	28 (90)	9 (100)	35 (81)
**Albania**	13 (65)	24 (77)	8 (89)	30 (70)
**Algeria**	14 (70)	27 (87)	9 (100)	33 (77)
**Antigua and Barbuda**	18 (90)	29 (94)	9 (100)	40 (93)
**Angola**	8 (40)	13 (42)	4 (44)	15 (35)
**Argentina**	19 (95)	30 (97)	9 (100)	41 (95)
**Armenia**	19 (95)	29 (94)	8 (89)	40 (93)
**Bahrain**	18 (90)	27 (87)	8 (89)	37 (86)
**Bangladesh**	17 (85)	28 (90)	8 (89)	37 (86)
**Barbados**	17 (85)	29 (94)	9 (100)	38 (88)
**Belarus**	15 (75)	26 (84)	9 (100)	33 (77)
**Belize**	19 (95)	30 (97)	9 (100)	41 (95)
**Bhutan**	18 (90)	30 (97)	8 (89)	40 (93)
**Bolivia**	19 (95)	28 (90)	9 (100)	39 (91)
**Bosnia and Herzegovina**	13 (65)	21 (68)	8 (89)	27 (63)
**Botswana**	17 (85)	30 (97)	9 (100)	39 (91)
**Brazil**	18 (90)	28 (90)	9 (100)	38 (88)
**Bulgaria**	13 (65)	20 (65)	9 (100)	25 (58)
**Burkina Faso**	16 (80)	30 (97)	9 (100)	38 (88)
**Burundi**	15 (71)	28 (90)	8 (89)	34 (79)
**Cambodia**	5 (25)	8 (26)	1 (11)	10 (23)
**Cameroon**	18 (90)	31 (100)	9 (100)	41 (95)
**Cape Verde**	20 (100)	31 (100)	9 (100)	43 (100)
**Central African Republic**	18 (90)	30 (97)	9 (100)	40 (93)
**Chad**	16 (80)	28 (90)	8 (89)	36 (84)
**Chile**	16 (80)	29 (94)	9 (100)	37 (86)
**China**	16 (80)	28 (90)	9 (100)	36 (84)
**Colombia**	18 (90)	28 (90)	9 (100)	38 (88)
**Congo**	18 (90)	28 (90)	8 (89)	38 (88)
**Cook Islands**	16 (80)	29 (94)	9 (100)	37 (86)
**Costa Rica**	17 (85)	28 (90)	9 (100)	37 (86)
**Côte d'Ivoire**	18 (90)	27 (87)	8 (89)	37 (86)
**Croatia**	19 (95)	29 (94)	9 (100)	40 (93)
**Cuba**	18 (90)	28 (90)	9 (100)	38 (88)
**Czech Republic**	18 (90)	29 (94)	9 (100)	39 (91)
**Democratic Peoples Republic of Korea**	17 (85)	28(90)	8 (89)	37 (86)
**Democratic Republic of Congo**	18 (90)	28 (90)	8 (89)	38 (88)
**Djibouti**	14 (70)	28 (90)	8 (89)	34 (79)
**Dominica**	18 (90)	29 (94)	9 (100)	39 (91)
**Dominican Republic**	20 (100)	29 (94)	9 (100)	41 (95)
**Ecuador**	17 (85)	28 (90)	8 (89)	38 (88)
**Egypt**	17 (85)	29 (94)	9 (100)	38 (88)
**El Salvador**	19 (95)	26 (84)	9 (100)	37 (86)
**Eritrea**	16 (80)	31 (100)	9 (100)	39 (91)
**Estonia**	14 (70)	22 (71)	7 (78)	29 (67)
**Ethiopia**	19 (95)	31 (100)	9 (100)	42 (98)
**Fiji**	18 (90)	29 (94)	9 (100)	39 (91)
**Gambia**	15 (75)	29 (94)	8 (89)	36 (84)
**Georgia**	19 (95)	28 (90)	8 (89)	39 (91)
**Ghana**	18 (90)	31 (100)	9 (100)	21 (95)
**Grenada**	18 (90)	29 (94)	9 (100)	39 (91)
**Guinea**	15 (75)	30 (97)	9 (100)	37 (86)
**Guyana**	18 (90)	28 (90)	9 (100)	38 (88)
**Haiti**	16 (80)	28 (90)	8 (89)	36 (84)
**Honduras**	18 (90)	26 (84)	9 (100)	36 (84)
**India**	18 (90)	28 (90)	9 (100)	38 (88)
**Indonesia**	18 (90)	28 (90)	9 (100)	38 (88)
**Iran (Islamic Republic of)**	20 (100)	29 (94)	9 (100)	41 (95)
**Iraq**	16 (80)	29 (94)	9 (100)	37 (86)
**Jamaica**	20 (100)	30 (97)	9 (100)	42 (98)
**Jordan**	19 (95)	30 (97)	9 (100)	41 (95)
**Kenya**	20 (100)	29 (94)	9 (100)	41 (95)
**Kiribati**	17 (85)	31 (100)	9 (100)	40 (93)
**Kyrgyzstan**	18 (90)	27 (87)	9 (100)	37 (86)
**Latvia**	17 (85)	19 (61)	8 (89)	28 (65)
**Lebanon**	18 (90)	28 (90)	8 (89)	38 (88)
**Lesotho**	15 (75)	26 (84)	8 (89)	33 (77)
**Liberia**	16 (80)	27 (87)	8 (89)	35 (81)
**Lithuania**	16 (80)	22 (71)	8 (89)	30 (70)
**Madagascar**	16 (80)	25 (81)	7 (78)	33 (77)
**Malawi**	16 (80)	31 (100)	9 (100)	39 (91)
**Malaysia**	18 (90)	28 (90)	9 (100)	38 (88)
**Maldives**	18 (90)	30 (97)	9 (100)	40 (93)
**Mali**	18 (90)	30 (97)	9 (100)	40 (93)
**Malta**	18 (90)	31 (100)	9 (100)	41 (95)
**Marshall Islands**	14 (70)	28 (90)	9 (100)	34 (79)
**Mauritania**	15 (75)	26 (84)	8 (89)	33 (77)
**Mexico**	20 (100)	28 (90)	9 (100)	40 (93)
**Mongolia**	18 (90)	29 (93)	9 (100)	39 (91)
**Montenegro**	19 (95)	25 (81)	9 (100)	36 (84)
**Morocco**	17 (85)	29 (94)	8 (89)	38 (88)
**Mozambique**	17 (85)	31 (100)	9 (100)	40 (93)
**Myanmar**	18 (90)	29 (94)	9 (100)	39 (91)
**Namibia**	17 (85)	31 (100)	9 (100)	40 (93)
**Nauru**	16 (80)	29 (94)	9 (100)	37 (86)
**Nepal**	19 (95)	29 (94)	9 (100)	40 (93)
**Nicaragua**	19 (95)	28 (90)	9 (100)	39 (91)
**Nigeria**	17 (85)	30 (97)	8 (89)	39 (91)
**Niue**	15 (75)	25 (81)	9 (100)	32 (74)
**Oman**	19 (95)	29 (94)	9 (100)	40 (93)
**Pakistan**	20 (100)	28 (90)	8 (89)	40 (93)
**Palau**	15 (75)	28 (90)	8 (89)	36 (84)
**Papua New Guinea**	18 (90)	30 (97)	9 (100)	40 (93)
**Paraguay**	18 (90)	27 (87)	8 (89)	37 (86)
**Peru**	19 (95)	30 (97)	9 (100)	41 (95)
**Philippines**	20 (100)	30 (97)	9 (100)	42 (98)
**Poland**	15 (75)	21 (68)	7 (78)	29 (67)
**Portugal**	20 (100)	30 (97)	9 (100)	42 (98)
**Republic of Moldova**	19 (95)	31 (100)	9 (100)	42 (98)
**Romania**	14 (70)	27 (87)	8 (89)	34 (79)
**Russian Federation**	17 (85)	27 (87)	9 (100)	36 (83)
**Rwanda**	15 (75)	29 (94)	8 (89)	36 (83)
**Saint Kitts and Nevis**	18 (90)	29 (94)	9 (100)	39 (91)
**Saint Lucia**	18 (90)	29 (94)	9 (100)	39 (91)
**Saint Vincent and the Grenadines**	19 (95)	30 (97)	9 (100)	41 (95)
**Senegal**	19 (95)	28 (90)	8 (89)	39 (91)
**Serbia**	18 (90)	26 (84)	8 (89)	37 (86)
**Seychelles**	17 (85)	27 (87)	8 (89)	36 (84)
**Slovakia**	19 (95)	30 (97)	9 (100)	41 (95)
**Slovenia**	20 (100)	27 (87)	9 (100)	39 (91)
**Solomon Islands**	20 (100)	29 (94)	9 (100)	41 (95)
**Somalia**	10 (50)	19 (61)	5 (56)	22 (51)
**South Africa**	17 (85)	29 (94)	9 (100)	38 (88)
**Sri Lanka**	17 (85)	22 (71)	9 (100)	31 (72)
**Sudan**	15 (75)	29 (94)	9 (100)	36 (84)
**Suriname**	19 (95)	28 (90)	9 (100)	39 (91)
**Sweden**	14 (70)	26 (84)	9 (100)	32 (74)
**Syrian Arab Republic**	19 (95)	31 (100)	9 (100)	42 (98)
**Tajikistan**	16 (80)	31 (100)	9 (100)	39 (91)
**Thailand**	19 (95)	29 (94)	9 (100)	40 (93)
**The former Yugoslav Republic of Macedonia**	16 (80)	27 (87)	8 (89)	35 (81)
**Timor-Leste**	17 (85)	30 (97)	9 (100)	39 (91)
**Togo**	17 (85)	30 (97)	9 (100)	39 (91)
**Tonga**	16 (80)	29 (94)	9 (100)	37 (86)
**Trinidad and Tobago**	19 (95)	29 (94)	9 (100)	40 (93)
**Tunisia**	17 (85)	29 (94)	9 (100)	38 (88)
**Tuvalu**	17 (85)	29 (94)	8 (89)	38 (88)
**Uganda**	20 (100)	29 (94)	8 (89)	41 (95)
**Ukraine**	20 (100)	30 (97)	9 (100)	42 (98)
**United Republic of Tanzania**	18 (90)	30 (97)	9 (100)	40 (93)
**Uruguay**	17 (85)	28 (90)	9 (100)	37 (86)
**Vanuatu**	15 (76)	29 (94)	8 (89)	36 (84)
**Venezuela (Bolivarian Republic of)**	18 (90)	29 (94)	9 (100)	39 (91)
**Viet Nam**	17 (85)	30 (97)	9 (100)	39 (91)
**Yemen**	18 (90)	26 (84)	8 (89)	36 (84)
**Zambia**	16 (80)	30 (97)	9 (100)	38 (88)
**Zimbabwe**	18 (90)	29 (94)	8 (89)	39 (91)
***Mean***	**17.1 (86)**	**27.9 (90)**	**8.5 (95)**	**37 (86)**
***Median (IQR)***	**90 (80–95)**	**94 (90–97)**	**100 (89–100)**	**88 (84–93)**

### Primary care priority interventions

The median percentage of countries that included each of the 31 interventions recommended in the PEN priority list was 94% (129 of 137) (IQR: 89–98%), that is, PEN priority medicines were included in most national EMLs (Table 4). The priority intervention included in the fewest national EMLs was senna (47 of 137 countries, 34%). The median percentage of PEN medicines included by each country was 94% (29 of 31) (IQR: 90–97%), that is, national EMLs included most priority medicines (Table 3). Thirteen countries had a coverage rate of 100%, meaning they included all 31 medicines or medicine classes on their EML (Table 3).

**Table 4:** Package of Essential Non-communicable (PEN) Disease Interventions for Primary Health care in Low-Resource Settings and the number of countries that listed them on their national EML.

Medicine	# of countries listing (%)
Thiazide Diuretic	134 (98)
Calcium Channel Blocker	135 (99)
Beta-blocker	136 (99)
Angiotensin inhibitor	134 (98)
Statin	111 (81)
Insulin	135 (99)
Metformin	133 (97)
Glibenclamide	122 (89)
Isosorbide dinitrate	119 (87)
Glyceryl trinitrate	120 (88)
Furosemide	133 (97)
Spironolactone	131 (96)
Salbutamol	135 (99)
Prednisolone	130 (95)
Beclomethasone	119 (87)
Acetylsalicylic acid	131 (96)
Acetaminophen (Paracetamol)	133 (97)
Ibuprofen	130 (95)
Codeine	94 (69)
Morphine	130 (95)
Penicillin	137 (100)
Erythromycin	126 (92)
Amoxicillin	137 (100)
Hydrocortisone	133 (97)
Epinephrine (Adrenaline)	128 (93)
Heparin	125 (91)
Diazepam	135 (99)
Magnesium	127 (93)
Promethazine	98 (72)
Senna (Sennosides)	47 (34)
Oxygen	89 (65)
***Median % (IQR)***	**95 (89–98)**

### Cardiovascular disease priority interventions

The median percentage of countries that included each of the 9 interventions recommended in the HEARTS document was 98% (134 of 137) (IQR: 96–99%), that is, HEARTS priority cardiovascular medicines were included in most national EMLs (Table 5). The median percentage of HEARTS medicines included by each country was 100% (IQR: 89–100%), that is, national EMLs included nearly all priority medicines (Table 3). A total of 92 countries had a coverage rate of 100%, listing all 9 interventions.

**Table 5:** HEARTS essential medicines recommendations and the number of countries that listed them on their national EML.

Medicine class/medicine	# of countries listing (%)
Angiotensin Blocker/Angiotensin converting enzyme inhibitor (ACEI)	134 (98)
Calcium channel blockers	135 (99)
Thiazide/ thiazide-like diuretics	134 (98)
Beta blockers	136 (99)
Metformin	133 (97)
Glibenclamide	122 (89)
Lipid-lowering therapy (statins)	111 (81)
Insulin	135 (99)
Acetylsalicylic acid	131 (96)
**MEDIAN % (IQR)**	**98 (96–99)**

### All priority NCD interventions

**Fig 2:**
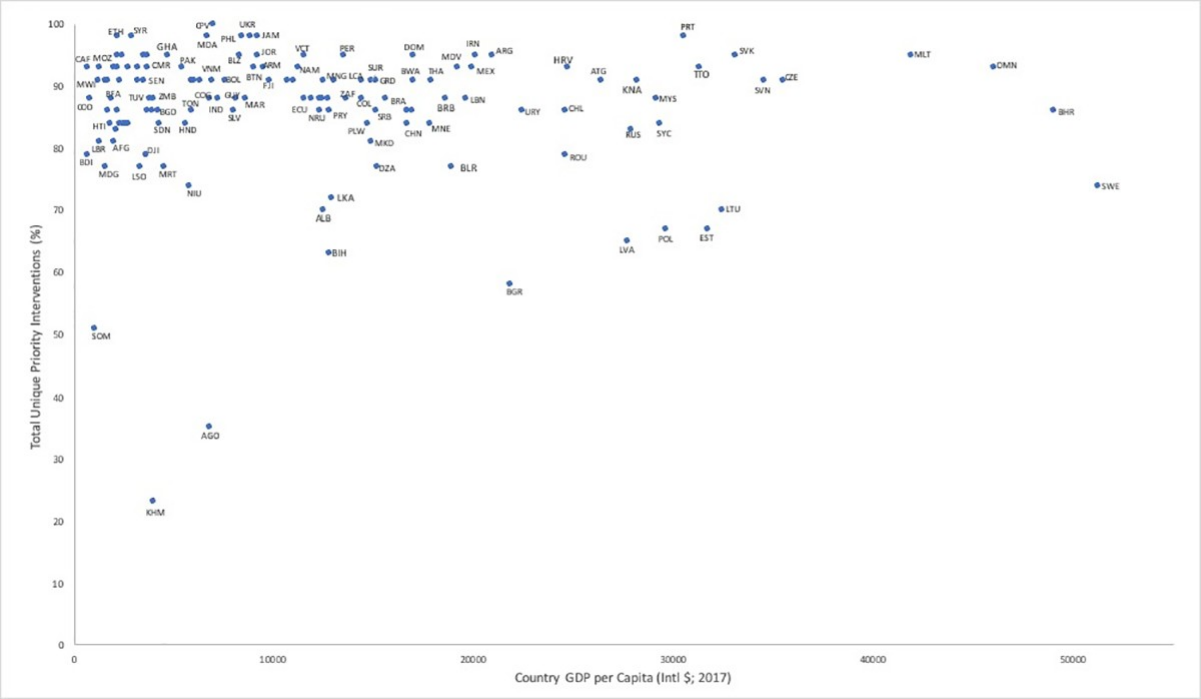
The relationship between percentage of unique interventions listed on NEMLs to country GDP.

The median percentage of countries that included each of the 43 unique interventions recommended through Best Buys, PEN and HEARTS was 88 (120 of 137) (IQR: 84–93%). Thus, most priority interventions were included by most countries (Table 3). Countries of high-income status included 65–98% of unique interventions; countries of upper-middle income included 58–98% of unique interventions; countries of lower-middle income included 23–100% of unique interventions; and countries of low-income status included 51–98% of recommended unique interventions (Fig 2). A total of 22 countries (16% of 137) listed fewer than 80% of the 43 interventions, and 2 countries listed below 50%: Angola (34.9%, 15 of 43) and Cambodia (23.3%, 10 of 43).

Priority interventions most commonly missing on national EMLs were: HPV vaccine, influenza vaccine, hepatitis B vaccine, cervical cancer chemotherapy, codeine, promethazine, senna, and oxygen [see S2 Table]. Of these, the three vaccines and cervical cancer chemotherapy were recommended in the Best Buys document; and codeine, promethazine, senna, and oxygen were recommended within PEN. Of all interventions examined, HPV vaccine was listed least frequently (22%) across 137 countries (Fig 1).

## Discussion

Listing a medicine as essential is an important first step toward ensuring its access; we found that most WHO priority NCD interventions are listed by most national EMLs. Only three priority interventions: HPV vaccination, influenza vaccination, and senna are listed by less than half of the countries. Three countries (Angola, Cambodia, and Somalia) list about half or less than half of priority interventions on any one of the WHO NCD priority packages.

### Comparison with previous studies

A previous study found that the proportion of countries listing medicines for secondary prevention of cardiovascular disease across 110 countries, ranges from low- to upper middle-income classification [6]. That study found, as we did, that beta blockers and ACE-inhibitors were included on most national EMLs whereas statins were listed less frequently [3]. Our study found that nearly all countries (96%, 131 of 137) included acetylsalicylic acid (aspirin) on their list (regardless of dose), while the previous study found a lower percentage of countries (67–69%) as listing aspirin (≤150 mg). We also found that nearly all countries (median 100%) included all 9 HEARTS medicines on their national EML (including 4 medicines for secondary prevention of cardiovascular disease), where only about half of countries examined in the other study included all four secondary prevention medicines [6]. Differences in the study approach were that our analysis did not compare EML listings based on country income classifications and included more recent versions of national EMLs as found in the WHO Repository (up to 2017, compared to up to 2015) [7, 16]. The differences in findings are thus likely explained by additional countries adding aspirin and statins to their national EML since 2015, our inclusion of a wider range of dosages, and the inclusion of high-income country EMLs in our study.

Another study looked at 24 cardiovascular disease medicines on the 2015 WHO EML listed on national EMLs in the WHO Eastern Mediterranean Region and found low percentages of these medicines listed in Djibouti (38%) and Tunisia (58%) [18]. Our study shows that these countries listed 89–100% of the 9 HEARTS medicines. Discrepancies between the two studies appear to be a result of methodological differences in investigating a specific medicine compared to a medication class, incorporating diverse dosage forms, or versions of national EMLs examined (e.g. the Tunisia and Bahrain EMLs are dated 2012 and 2015, respectively, in our study compared to a 2008 and 2009 in the other study).

The high percentages of countries listing the minimum medications required for diabetes management in our study are consistent with findings published in 2014 and 2016, which examined 32 LMICs and 25 countries across the Americas respectively [19, 20].

A 2014 audit surveyed 32 LMICs on their selection of medicines for asthma and COPD and found that over 90% of these countries had selected salbutamol and inhalation corticosteroid, primarily beclomethasone [21]. Our findings with a larger cohort of countries (137) were similar. Salbutamol was listed by 99% of countries, beclomethasone was listed by 87% of countries, and prednisolone, which was not included in the previous study, was listed by 95% of countries.

Previous studies on the listing of cancer medicines on national EMLs have primarily approached their analysis by looking at the percent overlap between the 38 cancer medicines listed on the 19^th^ WHO EML (2015) and national EMLs. Across 101 countries, a previous study found that most countries listed less than half of the WHO-recommended cancer medicines. The most similarity was identified between the WHO and the national EMLs in Iran (92%) and the least similarity in Colombia and Cambodia (2%) [22]. The study did not report findings based on type of cancer. A similar analysis in the WHO South-East Asia Region (SEARO) reported a mean of 18 (range 2–33) cancer medicines across national lists of 11 countries and found that the 6 most commonly listed medicines were bleomycin, cyclophosphamide, doxorubicin, vinblastine and vincristine plus cisplatin [23]. The most commonly listed chemotherapeutic agents across countries in our study were methotrexate (92% of 137 countries), cyclophosphamide (83%), 5-flurouracil (80%), doxorubicin (70%), irinotecan (68%), and cisplatin (66%).

The WHO Best Buys do not include all cancer medicines listed on the WHO EML but recommends “effective interventions with cost-effectiveness analysis ≤$100 per DALY averted in LMICs” for breast, colorectal, and cervical cancer [10]. Out of the chemotherapy regimens recommended, cervical cancer medicines were listed the least frequently by 66% of 137 countries compared to 96% and 85% for breast and colorectal cancer respectively. Our findings show room for improvement in the prioritization of cervical cancer prevention and treatment on national EMLs. As cervical cancer represents the leading cause of cancer death in 42 countries, primarily in sub‐Saharan Africa and South‐Eastern Asia, improving access to cervical cancer treatments (together with improvements in cervical cancer screening) appears especially prudent in these contexts [24].

Almost all countries have listed opiates for basic palliative care on their NEMLs. Although EML listing does not guarantee access to medicines, this finding is an encouraging beginning in the context of the current need to improve access to pain and palliative care, as 50% of the global population, primarily the world’s poorest, receive less than 1% of the worldwide morphine supply [25].

Two of the 3 WHO-recommended vaccines in the priority lists are most often missing from national EMLs. Of all interventions examined, the HPV vaccine was listed by the fewest number of countries. The 30 countries that did list HPV vaccine on their EML ranged from low- to high-income nations across diverse WHO regions. Omission of HPV vaccine from EMLs may reflect the relative newness of the intervention, however, it was introduced in 2006 [26] and the WHO recommends lists be updated every 2 years [27]. Studies have shown that HPV vaccination rates were much higher in more developed compared to less developed countries [28]. Over 85% of cervical cancer deaths occur in LMICs and only 14% of these countries are reported to have national HPV vaccination programs [29]. Diverse sociocultural, health system, financial and political challenges have been shown to influence the implementation of the HPV vaccine in LMICs [28–30]. Many solutions to these challenges have been proposed and put forward in the literature and by diverse global health actors [31–34]. Prioritization of the HPV vaccine on national EMLs could be an important first step toward encouraging rapid roll out of the vaccine and national programs aimed at reducing the preventable burden of cervical cancer.

Many of the same countries that did not list HPV vaccinations on their national EML also did not list the influenza vaccine. A 2017 study indicated that influenza vaccines were perhaps not suitable for prioritization in resource-limited settings and that they do not appear to meet WHO standards for programmatic suitability of vaccines [35]. This may in part explain why influenza vaccine has a lower priority on national EML lists, given that the majority of national EMLs examined in our study are LMIC lists.

### Strengths and limitations

The study strengths are that we were able to look at national EMLs across a large number of countries and that we were able to apply a global standard of basic care for NCDs by using the WHO-endorsed priority interventions for NCDs.

A limitation of this study is that the national lists analyzed may no longer be current, as we used the lists available on the WHO Repository around the time the study was conducted. The lists and the field of study are continuously evolving. Nevertheless, we used more recent EML versions than other published studies. Another limitation is that evidence is still relatively scarce on how national EMLs are used in different settings, such as their practical use in medicine procurement at national or subnational levels. The 3 priority lists of medicines we used are WHO-endorsed; if other lists were used, results may have differed. We did not investigate standard treatment guidelines or national budgetary allocation to medicines. Another limitation is that we looked at essential medicines for NCDs recommended by WHO on a global scale with attention to intervention cost-effectiveness, which may not necessarily be representative of the priority individual and population health needs in a given country. However, these medicines represent basic evidence-based priority interventions, largely for primary care, that are most likely applicable to investigate prioritization of NCD treatment in policy across diverse contexts.

## Conclusions and future work

There is a high degree of overlap between priority medicines for NCDs described by key WHO packages and those listed on national EMLs. The listing of priority NCD treatments on national EMLs signals an important first step toward making medicines accessible and moving toward realizing the WHO NCD targets in many countries. Our study also provides insight into national gaps and opportunities for improvement in prioritizing NCD medicines in different countries and across a range of priority medications, such as HPV vaccination. These findings can inform progress monitoring toward the Sustainable Development Goal on health. Importantly, the findings of NCD medicines listed or not listed on a given national EML offer actionable evidence for governments to revise their EMLs, or for advocacy and further research to understand national EML implementation where there are discrepancies between medicines listed and those accessible to those who need them. Advocates and researchers could urge or petition governments to either update their national EMLs to reflect context-specific population health needs. Future work should address how national EMLs are implemented in diverse contexts and further add to the sparse literature on the implications that national EML selection has on population access to these medicines across dimensions such as availability, affordability, and equity.

## Supporting information

S1 Table Country characteristics. (DOCX)

S2 Table Interventions missing from National Essential Medicines Lists. (DOCX)
