# Supplementary material for: Selection of WHO-recommended essential medicines for non-communicable diseases on National Essential Medicines Lists
Source: PLoS One. 2019 Aug 9;14(8):e0220781. doi: 10.1371/journal.pone.0220781 (PMC6688805; doi:10.1371/journal.pone.0220781)
Supplement: S1 Table — (DOCX) [file pone.0220781.s001.docx]

| **Country** | **Region** | **Population** | **Country GDP per Capita (Intl $; 2017)** | **Country NEML Year** | **Number of medications on list** |
| --- | --- | --- | --- | --- | --- |
| Afghanistan | Eastern Mediterranean | 34,124,811 | 2000 | 2014 | 260 |
| Albania | European | 3,047,987 | 12500 | 2011 | 215 |
| Algeria | Africa | 40,969,443 | 15200 | 2016 | 450 |
| Angola | Africa | 29,310,273 | 6800 | 2008 | 64 |
| Antigua and Barbuda | The Americas | 94,731 | 26400 | 2007 | 294 |
| Argentina | The Americas | 44,293,293 | 20900 | 2011 | 474 |
| Armenia | European | 3,045,191 | 9500 | 2010 | 271 |
| Bahrain (Kingdom of) | Eastern Mediterranean | 1,410,942 | 49000 | 2015 | 552 |
| Bangladesh | South-East Asia | 157,826,578 | 4200 | 2008 | 187 |
| Barbados | The Americas | 292,336 | 18600 | 2011 | 636 |
| Belarus | European | 9,549,747 | 18900 | 2012 | 372 |
| Belize | The Americas | 360,346 | 8300 | 2009 | 375 |
| Bhutan | South-East Asia | 758,288 | 9000 | 2016 | 294 |
| Bolivia | The Americas | 11,138,234 | 7600 | 2011 | 356 |
| Bosnia and Herzegovina | European | 3,856,181 | 12800 | 2009 | 182 |
| Botswana | Africa | 2,214,858 | 17000 | 2012 | 340 |
| Brazil | The Americas | 207,353,391 | 15600 | 2014 | 407 |
| Bulgaria | Europe | 7,101,510 | 21800 | 2011 | 362 |
| Burkina Faso | Africa | 20,107,509 | 1900 | 2014 | 274 |
| Burundi | Africa | 11,466,756 | 700 | 2012 | 295 |
| Cambodia | South-East Asia | 16,204,486 | 4000 | 2003 | 44 |
| Cameroon | Africa | 24,994,885 | 3700 | 2010 | 353 |
| Cabo (Cape) Verde | Africa | 560,899 | 7000 | 2009 | 567 |
| Central African Republic | Africa | 5,625,118 | 700 | 2009 | 295 |
| Chad | Africa | 12,075,985 | 2300 | 2007 | 241 |
| Chile | The Americas | 17,789,267 | 24600 | 2005 | 349 |
| China | Western Pacific | 1,379,302,771 | 16700 | 2012 | 292 |
| Colombia | The Americas | 47,698,524 | 14400 | 2011 | 373 |
| Congo | Africa | 4,954,674 | 6800 | 2013 | 303 |
| Cook Islands | Western Pacific | 9,290 | 16700 | 2007 | 240 |
| Costa Rica | The Americas | 4,930,258 | 16900 | 2014 | 389 |
| Côte d'Ivoire | Africa | 24,184,810 | 3900 | 2013 | 506 |
| Croatia | Europe | 4,292,095 | 24700 | 2010 | 603 |
| Cuba | The Americas | 11,147,407 | 12300 | 2012 | 511 |
| Czech Republic | Europe | 10,674,723 | 35500 | 2012 | 806 |
| Democratic Peoples Republic of Korea | South-East Asia | 51,181,299 | 1700 | 2012 | 221 |
| Democratic Republic of Congo | Africa | 83,301,151 | 800 | 2010 | 314 |
| Djibouti | Eastern Mediterranean | 865,267 | 3600 | 2007 | 201 |
| Dominica | The Americas | 73,897 | 11000 | 2007 | 285 |
| Dominican Republic | The Americas | 10,734,247 | 17000 | 2015 | 357 |
| Ecuador | The Americas | 16,290,913 | 11500 | 2013 | 369 |
| Egypt | Eastern Mediterranean | 97,041,072 | 12700 | 2012 | 324 |
| El Salvador | The Americas | 6,172,011 | 8000 | 2009 | 362 |
| Eritrea | Africa | 5,918,919 | 1600 | 2010 | 337 |
| Estonia | Europe | 1,251,581 | 31700 | 2012 | 405 |
| Ethiopia | Africa | 105,350,020 | 2200 | 2014 | 711 |
| Fiji | Western Pacific | 920,938 | 9800 | 2015 | 297 |
| Gambia | Africa | 2,051,363 | 2600 | 2001 | 165 |
| Georgia | Europe | 4,926,330 | 10700 | 2007 | 248 |
| Ghana | Africa | 27,499,924 | 4700 | 2010 | 303 |
| Grenada | The Americas | 111,724 | 15100 | 2007 | 284 |
| Guinea | Africa | 12,413,867 | 2200 | 2012 | 239 |
| Guyana | The Americas | 737,718 | 8100 | 2009 | 281 |
| Haiti | The Americas | 10,646,714 | 1800 | 2012 | 197 |
| Honduras | The Americas | 9,038,741 | 5600 | 2009 | 369 |
| India | South-East Asia | 1,281,935,911 | 7200 | 2015 | 368 |
| Indonesia | South-East Asia | 260,580,739 | 12400 | 2011 | 279 |
| Iran (Islamic Republic of) | Eastern Mediterranean | 82,021,564 | 20100 | 2014 | 896 |
| Iraq | Eastern Mediterranean | 39,192,111 | 16700 | 2010 | 577 |
| Jamaica | The Americas | 2,990,561 | 9200 | 2008 | 459 |
| Jordan | Eastern Mediterranean | 10,248,069 | 9200 | 2011 | 593 |
| Kenya | Africa | 47,615,739 | 3500 | 2016 | 417 |
| Kiribati | Western Pacific | 108,145 | 2000 | 2009 | 218 |
| Kyrgyzstan | Europe | 5,789,122 | 3700 | 2009 | 316 |
| Latvia | Europe | 1,944,643 | 27700 | 2012 | 308 |
| Lebanon | Eastern Mediterranean | 6,229,794 | 19600 | 2014 | 285 |
| Lesotho | Africa | 1,958,042 | 3300 | 2005 | 195 |
| Liberia | Africa | 4,689,021 | 1300 | 2011 | 215 |
| Lithuania | Europe | 2,823,859 | 32400 | 2012 | 340 |
| Madagascar | Africa | 25,054,161 | 1600 | 2008 | 253 |
| Malawi | Africa | 19,196,246 | 1200 | 2015 | 324 |
| Malaysia | Western Pacific | 31,381,992 | 29100 | 2014 | 310 |
| Maldives | South-East Asia | 392,709 | 19200 | 2009 | 539 |
| Mali | Africa | 17,885,245 | 2200 | 2012 | 285 |
| Malta | Europe | 416,338 | 41900 | 2008 | 612 |
| Marshall Islands | Western Pacific | 74,539 | 3600 | 2007 | 215 |
| Mauritania | Africa | 3,758,571 | 4500 | 2008 | 215 |
| Mexico | The Americas | 124,574,795 | 19900 | 2011 | 709 |
| Mongolia | South-East Asia | 3,068,243 | 13000 | 2009 | 258 |
| Montenegro | Europe | 642,550 | 17800 | 2011 | 453 |
| Morocco | Eastern Mediterranean | 33,986,655 | 8600 | 2012 | 344 |
| Mozambique | Africa | 26,573,706 | 1300 | 2016 | 259 |
| Myanmar (Burma) | South-East Asia | 55,123,814 | 6300 | 2010 | 315 |
| Namibia | Africa | 2,484,780 | 11200 | 2016 | 384 |
| Nauru | Western Pacific | 11,359 | 12300 | 2010 | 231 |
| Nepal | South-East Asia | 29,384,297 | 2700 | 2011 | 300 |
| Nicaragua | The Americas | 6,025,951 | 5900 | 2011 | 274 |
| Nigeria | Africa | 190,632,261 | 5900 | 2010 | 306 |
| Niue | Western Pacific | 1,618 | 5800 | 2006 | 215 |
| Oman | Eastern Mediterranean | 4,613,241 | 46000 | 2009 | 578 |
| Pakistan | Eastern Mediterranean | 204,924,861 | 5400 | 2016 | 374 |
| Palau | Western Pacific | 21,431 | 14700 | 2006 | 270 |
| Papua New Guinea | Western Pacific | 6,909,701 | 3700 | 2012 | 271 |
| Paraguay | The Americas | 6,943,739 | 12800 | 2009 | 307 |
| Peru | The Americas | 31,036,656 | 13500 | 2012 | 426 |
| Philippines | Western Pacific | 104,256,076 | 8400 | 2008 | 519 |
| Poland | Europe | 38,476,269 | 29600 | 2017 | 444 |
| Portugal | Europe | 10,839,514 | 30500 | 2011 | 909 |
| Republic of Moldova | Europe | 3,474,121 | 6700 | 2011 | 477 |
| Romania | Europe | 21,529,967 | 24600 | 2012 | 636 |
| Russian Federation | Europe | 142,257,519 | 27900 | 2014 | 520 |
| Rwanda | Africa | 11,901,484 | 2100 | 2010 | 288 |
| Saint Kitts and Nevis | The Americas | 52,715 | 28200 | 2006 | 291 |
| Saint Lucia | The Americas | 164,994 | 14400 | 2006 | 291 |
| Saint Vincent and the Grenadines | The Americas | 102,089 | 11500 | 2010 | 267 |
| Senegal | Africa | 14,668,522 | 3500 | 2013 | 339 |
| Serbia | Europe | 7,111,024 | 15100 | 2010 | 474 |
| Seychelles | Africa | 93,920 | 29300 | 2010 | 297 |
| Slovakia | Europe | 5,445,829 | 33100 | 20125 | 989 |
| Slovenia | Europe | 1,972,126 | 34500 | 2017 | 798 |
| Solomon Islands | Western pacific | 647,581 | 2200 | 2017 | 260 |
| Somalia | Africa | 11,031,386 | 1064* | 2006 | 83 |
| South Africa | Africa | 54,841,552 | 13600 | 2014 | 192 |
| Sri Lanka | South-East Asia | 22,409,381 | 12900 | 2013 | 318 |
| Sudan | Africa | 37,345,935 | 4300 | 2014 | 302 |
| Suriname | The Americas | 591,919 | 14900 | 2014 | 285 |
| Sweden | Europe | 9,960,487 | 51200 | 2016 | 289 |
| Syrian Arab Republic | Eastern Mediterranean | 18,028,549 | 2900 | 2008 | 974 |
| Tajikistan | Europe | 8,468,555 | 3200 | 2009 | 273 |
| Thailand | South-East Asia | 68,414,135 | 17900 | 2013 | 550 |
| The former Yugoslav Republic of Macedonia | Europe | 2,103,721 | 14900 | 2008 | 391 |
| Timor-Leste | South-East Asia | 1,291,358 | 6000 | 2015 | 240 |
| Togo | Africa | 7,965,055 | 1700 | 2012 | 297 |
| Tonga | Western Pacific | 106,479 | 5900 | 2007 | 229 |
| Trinidad & Tobago | The Americas | 1,218,208 | 31300 | 2010 | 495 |
| Tunisia | Eastern Mediterranean | 11,403,800 | 11900 | 2012 | 726 |
| Tuvalu | Western Pacific | 11,052 | 3800 | 2010 | 179 |
| Uganda | Africa | 39,570,125 | 2400 | 2012 | 365 |
| Ukraine | Europe | 44,033,874 | 8800 | 2009 | 280 |
| United Republic of Tanzania | Africa | 53,950,935 | 3200 | 2013 | 362 |
| Uruguay | The Americas | 3,360,148 | 22400 | 2011 | 527 |
| Vanuatu | Western Pacific | 282,814 | 2700 | 2006 | 177 |
| Venezuela (Bolivarian Republic of) | The Americas | 31,304,016 | 12500 | 2004 | 310 |
| Viet Nam | Western Pacific | 96,160,163 | 6900 | 2008 | 787 |
| Yemen | Eastern Mediterranean | 28,036,829 | 2500 | 2009 | 250 |
| Zambia | Africa | 15,972,000 | 4000 | 2013 | 288 |
| Zimbabwe | Africa | 13,805,084 | 2300 | 2011 | 347 |
