# Supplementary material for: Selection of WHO-recommended essential medicines for non-communicable diseases on National Essential Medicines Lists
Source: PLoS One. 2019 Aug 9;14(8):e0220781. doi: 10.1371/journal.pone.0220781 (PMC6688805; doi:10.1371/journal.pone.0220781)
Supplement: S2 Table — (DOCX) [file pone.0220781.s002.docx]

| **ATC Code** | **Intervention** | **Priority list** | **Countries not listing** |
| --- | --- | --- | --- |
| A06AB | Senna (Sennosides) | PEN | Afghanistan, Albania, Algeria, Antigua and Barbuda, Angola, Argentina, Bahrain, Barbados, Belarus, Belize, Bolivia, Bosnia and Herzegovina, Brazil, Bulgaria, Burundi, Cambodia, Chad, Chile, China, Colombia, Congo, Costa Rica, Côte d'Ivoire, Cuba, Czech Republic, Democratic Peoples Republic of Korea, Democratic Republic of Congo, Dominica, Dominican Republic, Ecuador, El Salvador, Estonia, Fiji, Grenada, Guyana, Haiti, Honduras, India, Indonesia, Iran (Islamic Republic of), Iraq, Jamaica, Kenya, Kyrgyzstan, Latvia, Lesotho, Liberia, Lithuania, Madagascar, Malaysia, Maldives, Mali, Marshall Islands, Montenegro, Morocco, Myanmar, Nauru, Nepal, Nicaragua, Niue, Oman, Palau, Paraguay, Peru, Poland, Romania, Rwanda, Saint Kitts and Nevis, Saint Lucia, Saint Vincent and the Grenadines, Senegal, Serbia, Seychelles, Slovakia, Solomon Islands, Somalia, Sudan, Suriname, Sweden, Timor-Leste, Tonga, Trinidad and Tobago, Tunisia, Tuvalu, Uganda, United Republic of Tanzania, Uruguay, Vanuatu, Viet Nam, Yemen |
| A10A (B, C, D, E) | Insulin | Best buys, PEN, HEARTS | Angola, Cambodia |
| A10B | Drug therapy including glycaemic control for diabetes mellitus (any oral hypoglycemic) | Best Buys | Angola |
| A10BA | Metformin | PEN, HEARTS | Angola, Cambodia, Madagascar, Somalia |
| A10BB | Glibenclamide | PEN, HEARTS | Angola, Bahrain, Bhutan, Cambodia, Côte d'Ivoire, Estonia, Latvia, Lebanon, Lithuania, Morocco, Pakistan, Paraguay, Poland, Senegal, Seychelles |
| A12CC | Magnesium | PEN | Albania, Bosnia and Herzegovina, Cambodia, Estonia, Honduras, Latvia, Lithuania, Poland, Romania, The former Yugoslav Republic of Macedonia |
| B01A | Anticoagulation for medium-and high-risk non-valvular atrial fibrillation and for mitral stenosis with atrial fibrillation | Best Buys | Afghanistan, Algeria, Angola, Brazil, Burundi, Cambodia, Chad, Chile, Côte d'Ivoire, Guinea, Lebanon, Madagascar, Mali, Mauritania, Morocco, Niue, Romania, Rwanda, Senegal, Somalia, South, Suriname, The former Yugoslav Republic of Macedonia, Togo, Tunisia, Zambia |
| B01AB | Heparin | PEN | Albania, Angola, Bulgaria, Burkina Faso, Cambodia, Estonia, Gambia, Latvia, Lithuania, Niue, Poland, Somalia |
| B01AC | Acetylsalicylic acid | Best Buys, PEN, HEARTS | Albania, Bosnia and Herzegovina, Estonia, Poland, Romania, Serbia |
| B01AD | Treatment of acute ischemic stroke with intravenous thrombolytic therapy (streptokinase) | Best Buys | Afghanistan, Albania, Algeria, Bahrain, Bangladesh, Belarus,  Bosnia and Herzegovina, Bulgaria, Burkina Faso, Burundi, Cambodia, Chad, China, Congo, Czech Republic, Democratic Peoples Republic of Korea, Democratic Republic of Congo, Djibouti Eritrea, Estonia, Ethiopia, Gambia, Haiti, Iraq, Latvia, Lesotho, Liberia, Lithuania, Madagascar, Malawi, Malaysia, Malta, Marshall Islands, Mongolia, Mozambique, Namibia, Nigeria, Niue, Poland, Romania, Rwanda, Seychelles, Slovakia, Somalia, Sweden, Tajikistan, Tunisia, Vanuatu, Zambia |
| C01CA | Epinephrine (Adrenaline) | PEN | Albania, Belarus, Bosnia and Herzegovina, Bulgaria, Cambodia, Estonia, Latvia, Lithuania, Sri Lanka |
| C01DA | Glyceryl trinitrate | PEN | Angola, Brazil, Bulgaria, Burundi, Cambodia, Congo, Democratic Republic of Congo, Djibouti, Estonia, Latvia, Madagascar, Mauritania, Seychelles, Slovenia, Somalia, South Africa, Suriname |
| C01DA | Isosorbide dinitrate | PEN | Angola, Cambodia, Central African Republic, Chad, Côte d'Ivoire, Honduras, Liberia, Malaysia, Marshall Islands, Montenegro, Nauru, Niue, Poland, Slovenia, Somalia, Sri Lanka, Sweden, Zambia |
| C03AA | Diuretic | Best Buys, PEN, HEARTS | Cambodia, Ecuador, Palau |
| C03DA | Spironolactone | PEN | Angola, Cambodia, Croatia, Niue, Sri Lanka, Yemen |
| C07A | Beta-blocker | Best buys, PEN, HEARTS | Cambodia |
| C08 (C, D) | Calcium Channel Blocker | PEN, HEARTS | Cambodia, Somalia |
| C09AA | Angiotensin converting enzyme inhibitor | Best buys, PEN, HEARTS | Angola, Cambodia, Somalia |
| C10AA | Statin | PEN, HEARTS | Angola, Armenia, Bangladesh, Burundi, Cambodia, Chad, Congo, Democratic Peoples Republic of Korea, Democratic Republic of Congo, Djibouti, Gambia, Georgia, Haiti, Lesotho, Liberia, Madagascar, Mauritania, Nigeria, Rwanda, Somalia, The former Yugoslav Republic of Macedonia, Tuvalu, Uganda, Vanuatu, Yemen, Zimbabwe |
| H02AB | Hydrocortisone | PEN | Albania, Angola, Bosnia and Herzegovina, Cambodia |
| J01FA | Erythromycin | PEN | Angola, Bulgaria, Costa Rica, El Salvador, Kyrgyzstan, Latvia, Mongolia, Pakistan, Paraguay, Poland, Russian Federation |
| J07BB | Influenza Vaccination* | Best Buys | Afghanistan, Albania, Antigua and Barbuda, Angola, Bangladesh, Barbados, Belarus, Bhutan, Bosnia and Herzegovina, Bostwana, Bulgaria, Burkina Faso, Burundi, Cambodia, Cameroon, Central African Republic, Chad, Chile, China, Colombia, Congo, Cook Islands, Costa Rica, Cuba, Democratic Peoples Republic of Korea, Democratic Republic of Congo, Djibouti, Dominica, Ecuador, Egypt, Eritrea, Estonia, Fiji, Gambia, Ghana, Grenada, Guinea, Guyana, Haiti, Honduras, India, Indonesia, Iraq, Kiribati, Lesotho, Liberia, Lithuania, Madagascar, Malawi, Mali, Marshall Islands, Mauritania, Morocco, Mozambique, Myanmar, Namibia, Nauru, Nigeria, Papua New Guinea, Paraguay, Peru, Poland, Romania, Russian Federation, Rwanda, Saint Kitts and Nevis, Saint Lucia, Seychelles, Somalia, Sri Lanka, Sudan, Sweden, Tajikistan, The former Yugoslav Republic of Macedonia, Timor-Leste, Togo, Tonga, Tuvalu, United Republic of Tanzania, Uruguay, Vanuatu, Venezuela (Bolivarian Republic of), Viet Nam, Yemen, Zambia, Zimbabwe |
| J07BC | Prevention of liver cancer through hepatitis B immunization | Best Buys | Albania, Algeria, Angola, Barbados, Belarus, Bosnia and Herzegovina, Bulgaria, Cambodia, Chile, China, Costa Rica, Czech Republic, Estonia, Iraq, Latvia, Lesotho, Lithuania, Malawi, Marshall Islands, Poland, Romania, Russian Federation, Somalia, Sudan, Sweden, The former Yugoslav Republic of Macedonia, Uruguay, Viet Nam |
| J07BM | Vaccination against human papillomavirus | Best Buys | Afghanistan, Albania, Algeria, Antigua and Barbuda,  Angola,  Argentina,  Armenia, Bahrain, Bangladesh, Barbados, Belarus, Belize, Bolivia, Bosnia and Herzegovina, Bostwana, Bulgaria, Burkina Faso, Burundi, Cambodia, Cameroon, Central African Republic, Chad, Chile, China, Colombia, Cook Islands, Costa Rica, Côte d'Ivoire, Croatia, Cuba, Democratic Peoples Republic of Korea, Djibouti, Dominica, Ecuador, Egypt, El Salvador, Eritrea, Estonia, Gambia, Georgia, Ghana, Grenada, Guinea, Guyana, Haiti, Honduras, India, Indonesia, Jordan, Kiribati, Kyrgyzstan, Latvia, Lebanon, Lesotho, Liberia, Lithuania, Madagascar, Malawi, Maldives, Malta, Marshall Islands, Mauritania, Mongolia, Montenegro, Morocco, Namibia, Nauru, Nepal, Nicaragua, Nigeria, Niue, Oman, Palau, Papua New Guinea, Paraguay, Poland, Republic of Moldova, Romania, Russian Federation, Rwanda, Saint Kitts and Nevis, Saint Lucia, Saint Vincent and the Grenadines, Serbia, Seychelles, Somalia, Sri Lanka, Sudan, Sweden, Syrian Arab Republic, Tajikistan, Thailand, The former Yugoslav Republic of Macedonia, Timor-Leste, Togo, Tonga, Trinidad and Tobago, Tunisia, Tuvalu, United Republic of Tanzania, Uruguay, Vanuatu, Venezuela (Bolivarian Republic of), Viet Nam, Yemen, Zambia, Zimbabwe |
| L01 (A, B, C, D, X) * | Colorectal cancer chemotherapy | Best Buys | Albania, Algeria, Angola, Bosnia and Herzegovina, Bulgaria, Burundi, Cambodia, Cook Islands, Djibouti, Gambia, Guinea, Marshall Islands, Mauritania, Nauru, Niue, Palau, Somalia, Sudan, Sweden, Tonga, Vanuatu |
| L01 (A, B, C, D, X)* | Cervical Cancer Chemotherapy | Best Buys | Afghanistan, Albania, Algeria, Angola, Belarus, Bhutan, Bosnia and Herzegovina, Bostwana, Brazil, Bulgaria, Burkina Faso, Burundi, Cambodia, Cook Islands, Djibouti, Egypt, Eritrea, Estonia, Fiji, Gambia, Guinea, Haiti, Iraq, Kiribati, Kyrgyzstan, Lesotho, Liberia, Malaysia, Maldives, Marshall Islands, Mauritania, Mozambique, Myanmar, Nauru, Niue, Palau, Rwanda, Somalia, South Africa, Sudan, Sweden, Tajikistan, Timor-Leste, Tonga, Tuvalu, Vanuatu |
| L01 (A, B, C, D, X)* | Breast Cancer Chemotherapy | Best Buys | Angola, Cambodia, Djibouti, Palau, Somalia, South Africa |
| M01AE | Ibuprofen | PEN | Angola, Cambodia, Latvia, Mexico, Myanmar, Papua New Guinea, Suriname |
| N02A (A, B, C, D, E, F)* | Opiates  for Basic palliative care for cancer | Best Buys | Angola, Bulgaria, Cambodia, Somalia |
| N02AA | Morphine | PEN | Algeria, Angola, Bosnia and Herzegovina, Bulgaria, Cambodia, Somalia, Sri Lanka |
| N02BE | Acetaminophen | PEN | Bulgaria, Latvia, Lithuania, Madagascar |
| N05BA | Diazepam | PEN | Bulgaria, Sri Lanka |
| R01AD | Prednisolone | PEN | Angola, Bolivia, Bosnia and Herzegovina, China, Lesotho, Seychelles, Tonga |
| R03AC | Salbutamol | Best Buys, PEN | Cambodia, Sri Lanka |
| R03AC, R03BA | Treatment of asthma using low dose inhaled beclomethasone AND a short acting beta agonist | Best Buys | Afghanistan, Angola, Bangladesh, Bosnia and Herzegovina, Cambodia, Côte d'Ivoire, Democratic Peoples Republic of Korea, Georgia, India, Indonesia, Lesotho, Mauritania, Montenegro, Senegal, Somalia, Sri Lanka, Sweden, Uruguay, Yemen |
| R03BA | Beclomethasone | PEN | Afghanistan, Angola, Bangladesh, Bosnia and Herzegovina, Cambodia, Côte d'Ivoire, Democratic Peoples Republic of Korea, Georgia, India, Indonesia, Lesotho, Mauritania, Montenegro, Senegal, Somalia, Sweden, Uruguay, Yemen |
| R05DA | Codeine | PEN | Afghanistan, Angola, Armenia, Bahrain,  Bangladesh, Belarus, Bulgaria, Cambodia, Colombia, Cook Islands, Djibouti, Dominica, Egypt, El Salvador, Georgia, Grenada, Haiti, India, Kyrgyzstan, Latvia, Lebanon, Lesotho, Liberia, Lithuania, Madagascar, Malaysia, Mauritania, Montenegro, Nepal, Nicaragua, Niue, Pakistan, Poland, Russian Federation, Saint Kitts and Nevis, Saint Lucia, Serbia, Somalia, South Africa, Sri Lanka, Sudan, Ukraine, Yemen |
| R06AD | Promethazine | PEN | Algeria, Belarus, Bolivia, Bosnia and Herzegovina, Bulgaria, Chile, Colombia, Cook Islands, Croatia, Cuba, Dominican Republic, Ecuador, Egypt, El Salvador, Estonia, Guinea, Guyana, Honduras, Indonesia, Kyrgyzstan, Latvia, Lithuania, Mauritania, Mexico, Mongolia, Montenegro, Nicaragua, Paraguay, Philippines, Poland, Russian Federation, Serbia, Slovenia, Sri Lanka, Sweden, Thailand, The former Yugoslav Republic of Macedonia, Togo, Venezuela (Bolivarian Republic of) |
| V03AN | Oxygen | PEN | Albania, Algeria, Angola, Bahrain, Barbados, Belarus, Bosnia and Herzegovina, Bostwana, Brazil, Bulgaria, Cambodia, China, Costa Rica, Cuba, Czech Republic, El Salvador, Estonia, Fiji, Guyana, Honduras, Iran (Islamic Republic of), Iraq, Jordan, Kenya, Latvia, Lebanon, Lithuania, Marshall Islands, Mexico, Montenegro, Niue, Oman, Palau, Poland, Portugal, Romania, Russian Federation, Serbia, Slovenia, Solomon Islands, Somalia, Sweden, Thailand, The former Yugoslav Republic of Macedonia, Trinidad and Tobago, Tunisia, Uruguay, Venezuela (Bolivarian Republic of) |
